# Trajectories of psychosocial working conditions and all-cause and cause-specific mortality: a Swedish register-based cohort study

**DOI:** 10.5271/sjweh.4111

**Published:** 2023-10-01

**Authors:** Kuan-Yu Pan, Melody Almroth, Alicia Nevriana, Tomas Hemmingsson, Katarina Kjellberg, Daniel Falkstedt

**Affiliations:** 1 Unit of Occupational Medicine, Institute of Environmental Medicine, Karolinska Institutet, Stockholm, Sweden.; 2 Department of Public Health Sciences, Stockholm University, Stockholm, Sweden.; 3 Centre for Occupational and Environmental Medicine, Region Stockholm, Stockholm, Sweden.

**Keywords:** alcohol-related, all-cause mortality, death, cardiovascular disease, group-based trajectory modelling, job control, job demand, job demand-control, suicide

## Abstract

**Objectives:**

While psychosocial working conditions have been associated with morbidity, their associations with mortality, especially cause-specific mortality, have been less studied. Additionally, few studies considered the time-varying aspect of exposures. We aimed to examine trajectories of job demand–control status in relation to all-cause and cause-specific mortality, including cardiovascular diseases (CVD), suicide, and alcohol-related mortality.

**Methods:**

The study population consisted of around 4.5 million individuals aged 16-60 years in Sweden in 2005. Job control and demands were respectively measured using job exposure matrices (JEM). Trajectories of job control and demands throughout 2005–2009 were identified using group-based trajectory modelling, and job demand–control categories were subsequently classified. Deaths in 2010–2019 were recorded in the national cause of death register. Cox regression models were used.

**Results:**

A total of 116 242 individuals died in 2010–2019. For both job control and demands, we identified four trajectories, which were parallel to each other and represented four levels of exposures. Low control and passive jobs were associated with higher all-cause, CVD, and suicide mortality among both men and women. High strain jobs were associated with higher all-cause and CVD mortality among men, while low control, passive jobs, and high strain jobs were associated with higher alcohol-related mortality among women.

**Conclusions:**

The trajectories identified may suggest stable levels of job control and demands over time. Poor psychosocial working conditions are related to all-cause and cause-specific mortality, and these patterns vary to some extent between men and women.

Psychosocial working conditions pertain to the organization of work and interpersonal and social interactions at work that influence workers’ behavior and development in the work environment (1). Considering the amount of time in life an individual spends at work, exposure to adverse psychosocial working conditions has been suggested to cause health problems. In the past decades, mounting evidence has linked psychosocial working conditions to various health consequences, and some of the more established associations concerned cardiovascular diseases (CVD) and mental and behavioral disorders (2, 3). CVD (4) and mental and behavioral disorders (5) pose a great burden to societies worldwide. In Sweden they are among the leading causes of death (6). However, evidence relating psychosocial working conditions to mortality, especially mortality from CVD and mental and behavioral disorders, such as suicide and alcohol-related morbidity, has been relatively limited and inconclusive.

Among existing theoretical models, the job demand–control model (7) has been widely applied for the assessment of psychosocial working conditions. The demand dimension refers to psychological demands, the amount of work, and time restriction. Job control, comprising decision authority and skill discretion, refers to the extent to which a worker has influence in making decisions about the way the work is done, the level of monotony and skill utilization, and the possibility to develop in the occupation. Jobs that have high demands in combination with low control are termed as high strain jobs and deemed a stressful work environment. Passive jobs, where demands and control are both low, can demotivate workers because of underutilization of their abilities and loss of self-efficacy.

A systematic review and meta-analysis reported that low job control was associated with a higher risk of all-cause and coronary heart disease mortality but, despite being in the same direction, the association for high strain jobs was not statistically significant (8). However, there existed heterogeneity across studies included in the pooled results, making it challenging to draw conclusions from the meta-analysis.

Similarly, another systematic review and meta-analysis found low job control, but not job demands or high strain jobs, to be associated with a higher risk of suicide mortality (9). This review, however, underscored some important methodological limitations in the studies included, particularly the lack of longitudinal study design.

Most previous studies on the association between job control, job demands, and all-cause and cause-specific mortality used self-reported measures of job exposures, which may be subject to reporting bias. Few studies assessed both job control and demands using job-exposure matrices (JEM), which would reduce such bias because JEM typically are constructed based on national surveys on work environment and provide assessments of exposures on the occupational level. Additionally, most studies relied on a single measure of job exposures at baseline and not on integrated measures of exposures over periods of time. The lack of repeated measures of job exposures within individuals may result in a higher probability of misclassification and miss the opportunity to evaluate long-term, cumulative effects of exposures (10).

Recently, a series of French studies (the STRESSJEM study) retrospectively constructed three time-varying measures (current, cumulative, and recency-weighted cumulative exposures) of both job control and demands of all jobs held during 1976–2002 using JEM in relation to mortality over the same period (10). They found that low control, passive jobs, and high strain jobs were associated with a higher risk of all-cause mortality and mortality from CVD and alcohol-related morbidity among both men and women, and that the results were similar across the three time-varying measures. The association between job demands and these outcomes nevertheless did not manifest a consistent pattern across models (11–13). Additionally, they found low job control, passive jobs, and high strain jobs to be related to higher suicide mortality among men (14). However, considering that the data in these studies were collected more than two decades ago, it is unknown to what extent these results can be generalized to today’s working population.

In the current study, we utilized an occupational register that covers all jobs held between 2005 and 2009 and assessed both job control and demands of each job using JEM. We aimed to (i) explore the trajectories of job control and demands and their combinations over these years and (ii) investigate their prospective associations with all-cause mortality and mortality from CVD, suicide, and alcohol-related morbidity. We hypothesized that persons who have exposures to lower job control, passive jobs, or high strain jobs have a higher risk of all-cause, CVD, suicide, and alcohol-related mortality.

## Methods

### Study population

This study was based on the Swedish Work, Illness, and labor-market Participation (SWIP) cohort which includes all (around 5.4 million) individuals aged 16–65 years and registered in Sweden during the baseline year of 2005. Data were retrieved from several Swedish administrative and medical registers, including the Swedish total population register (15), the longitudinal integrated database for health insurance and labor market studies (LISA) register (16), the Swedish national patient register (17), the Swedish cause of death register (18), as well as information from earlier population censuses. Using personal identification numbers, Statistics Sweden made linkages between registers.

The present study included those who were aged 16–60 years in 2005, were alive before 2010, and had ≥1 year of information on job held between 2005 and 2009 to allow for the assessment of occupational exposures. We excluded those aged >60 years in 2005 because they were close to retirement age and thus information on job exposure after 2005 was limited. This resulted in a final population of 4 458 673, of which 49.8% were women.

Ethical approval was obtained by the Regional Ethics Review Board in Stockholm reference number 2017/1224-31 and 2018/1675-32.

### Exposures

We constructed the exposure variables in two steps.

In the first step, we measured job control and job demands using the Swedish JEM, which were constructed based on the Swedish Work Environment Surveys (1997–2013). These survey responses are aggregated for around 350 occupations, separately for men and women. We linked the JEM to the study population using occupational codes in the LISA register (16) for each individual yearly between 2005 and 2009 based on the Swedish ISCO-88 four-digit classification of occupations.

Job control was measured using four questions concerning decision latitude and four questions concerning skill discretion. Job demands were measured using three questions. The translated items are shown in the supplementary material www.sjweh.fi/article/4111, table S1, and extensive information on the construction of these JEM is described in detail elsewhere (19). These measures were scored as a mean for each occupation (range 1–10); a higher score of job control indicates a higher level of job control, while a higher score of job demands indicates a lower level of job demands.

In the second step, we constructed trajectories of both job control and demands, respectively for men and women, using group-based trajectory modelling (GBTM) (20, 21). In identifying trajectories/groups, we first entered continuous scores of job control and demands from 2005 to 2009 for each individual into the dataset. Next, we chose specific models, including type of model, shape of trajectories, and number of groups. Because job control and demands were continuous variables, we used censored normal regression models. With one measure of job control and demands yearly from 2005 to 2009, yielding five time points, we tested a quadratic shape model for all groups, and the quadratic component was statistically significant (P<0.01). We compared three models with three, four and five groups for job control and demands, respectively. Finally, following the previous suggestions (20, 21), the final number of groups was determined based on the Bayesian Information Criterion (BIC). BIC values closer to zero denote a better fitting model. For model diagnostics, we assessed the models using the following criteria (20, 21): (i) sufficient sample size in each identified group (>5%), (ii) close correspondence between the proportion estimated from the model and the proportion of individuals classified in such group according to the rule of attribution of the maximum probability of belonging, (iii) average posterior probability of assignment ≥0.7 for each group, and (iv) odds of correct classification based on the posterior probabilities of group membership >5.0. These diagnostics are shown in supplementary table S2.

**Figure 1:**
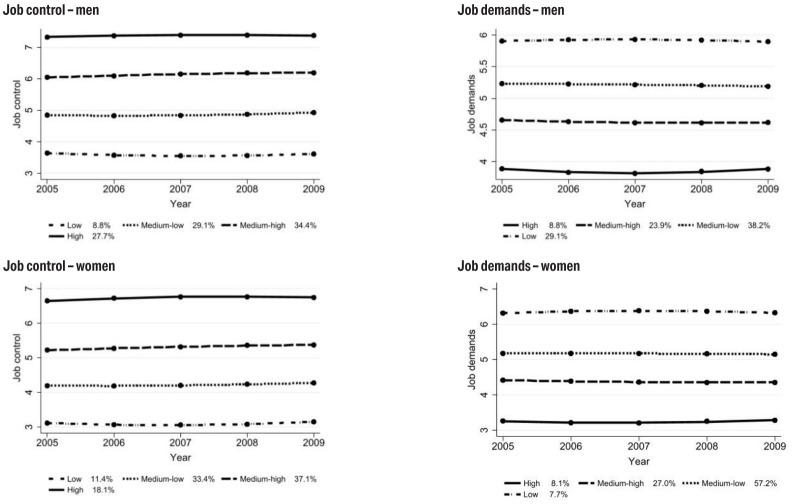
Trajectories of job control and job demands (2005–2009) in the Swedish working population by sex. A higher score of job control means a higher level of job control; A higher score of job demands means a lower level of job demands.

We identified four trajectories of job control and four trajectories of job demands from 2005 to 2009, separately for men and women (figure 1). These trajectories were parallel to each other, meaning that there was no group >5% that consisted of individuals who substantially changed the levels of job control or job demands over the years. Similar patterns were also seen among individuals <30 years in 2005 (supplementary figure S1). We respectively dichotomized job control and demands by grouping low and medium-low as low level and medium-high and high as high level. Subsequently, we classified four job strain categories: low strain jobs with low demands and high control, passive jobs with low demands and low control, active jobs with high demands and high control, and high strain jobs with high demands and low control.

### Outcomes

We used the Swedish cause of death register (18) to identify deaths between 2010 and 2019. We identified cause-specific deaths according to ICD-10 diagnosis codes: CVD, codes I00-I99; suicide, codes X60-X84, Y10-Y34; and alcohol-related morbidity, codes F10, K70, T51, X65, Y90, Y91, E24.4, G31.2, G62.1, G72.1, I42.6, K29.2, K85.2, K86.0, O35.4, R78.0, Y57.3, Z50.2, Z71.4, Z72.1 (22).

### Covariates

Age and sex were obtained from the total population register and the remaining covariates were taken from the LISA register in 2005. Birth country was categorized according to whether the individual was born in Sweden or not. Highest attained education was categorized as (i) primary and lower secondary school or less (≤9 years); (ii) secondary (10–11 years); (iii) upper-secondary (12 years); (iv) ≤2 years of post-secondary/university (13–15 years); and (v) >3 years of post-secondary/university (>15 years). Civil status was categorized as married/partnered, unmarried, divorced and widowed. Number of children was categorized as 0, 1–2, and ≥3 children.

Long-term sick leave during the five years prior to baseline was reported in the LISA register and defined as a period of >300 days in a calendar year. Histories of somatic disorders and psychiatric disorders were obtained using ICD-10, ICD-9, and ICD-8 codes from inpatient and outpatient registers from 1973 onward and prior to baseline. Somatic disorders included all ICD-10 codes, except F, O, P, Q codes. Psychiatric disorders included ICD-10 codes F01-F99. CVD, suicide attempt, and alcohol-related morbidity were identified using the same ICD-10 codes outlined in the outcomes section. Corresponding ICD-8 and ICD-9 codes are listed in supplementary table S3.

Individuals were linked to their parents; we used information from the population and housing censuses from 1960 (for those born 1941–1954), 1970 (for those born 1955–1964), 1980 (for those born 1965–1974) and 1990 (for those born 1975–1989) to capture their early-life socioeconomic position (SEP). This was estimated according to the father’s occupation, or mother’s occupation if father’s was missing, and categorized as non-manual employees at a higher level, non-manual employees at an intermediate level, assistant non-manual employees, skilled manual workers, non-skilled manual workers, farmers, and those with no parental occupation documented. We also obtained parents’ age at death. Early death of parents was defined as father or mother who died before the age of 65.

### Statistical analysis

Baseline characteristics of the study population were explored according to the outcome by the end of follow-up period.

Cox proportional hazard regression models with age as the underlying timescale were built to estimate hazard ratios (HR) and 95% confidence intervals (CI) for the associations of trajectories of job control and job demands and job strain categories with all-cause and cause-specific mortality. According to the strain hypothesis of the demand–control model, low strain was used as the reference category for all outcomes (7). Person-time was counted from 1 January 2010 until death, emigration, or the end of the follow-up period on 31 December 2019, whichever came first. When looking at a specific cause of death, persons who died due to other causes were censored on the date of death.

The effect modification by sex was tested by entering an interaction term of job exposure variable and sex in the model. The likelihood ratio test indicated that there were sex differences in almost all the combinations of exposure and outcome variables (P<0.05). Therefore, all models were run for men and women separately.

Model 1 was adjusted for age. Model 2 was further adjusted for birth year, birth country, civil status, number of children, early-life SEP, previous long-term sick leave for all outcomes, and additionally early death of parents for all-cause, CVD and alcohol-related mortality; history of somatic disorders for all-cause mortality; history of psychiatric disorders for all-cause and suicide mortality; history of CVD for CVD mortality; history of suicide attempt for suicide mortality; and history of alcohol-related morbidity for alcohol-related mortality (supplementary table S4). Finally, model 3 was additionally adjusted for education.

We did not test the proportional hazards assumption because our aim was to estimate the weighted average HR over time in our study population. However, age was the underlying timescale of the models. Considering that suicide deaths seemed to be much more evenly distributed across age groups than the other causes of death, we explored potential effect modification by age by stratified analyses in four age groups at baseline (16–29, 30–39, 40–49 and 50–60) for suicide mortality.

The identified trajectories showed stable levels of job control and job demands over the years, suggesting that job exposures in one year may be a proxy for the time-varying or long-term effect of exposures. We therefore reran analyses using only job exposures in 2009.

Data management and statistical analyses were done using STATA 17 (StataCorp LLC, College Station, TX, USA.)

## Results

During 2010 and 2019, there were 116 242 deaths in the study population. Of them, 25 242 died from CVD, 7314 from suicide, and 3001 from alcohol-related morbidity. Individuals who died during the follow-up period, compared to those who did not, were older, more likely to be men, married or divorced, to have lower education, previous long-term sick leave, and a history of somatic or psychiatric disorders, CVD, suicide attempt, or alcohol morbidity. They were less likely to have children, and their parents were less likely to be non-manual workers at a higher level (table 1).

**Table 1:** Baseline characteristics of study population (N=4 458 673) according to the outcome by the end of follow-up on 31 December 2019. [CVD=cardiovascular diseases; AR=alcohol-related.]

Characteristics		Died								
		No		Yes						
				All-cause (N=116 242)		CVD (N=25,242)		Suicide (N=7314)		AR (N=3001)
		%		%		%		%		%
Age (years)										
	16–29	27.2		4.7		1.5		24.2		1.6
	30–39	25.2		8.4		6.0		23.0		9.5
	40–49	24.0		21.4		20.9		28.6		28.6
	50–60	23.6		65.5		71.6		24.2		60.3
Women		50.1		40.5		27.7		29.7		27.8
Foreign born		12.4		11.9		11.8		10.9		11.4
Education years										
	≤9	16.8		25.0		28.0		24.2		27.7
	10–11	25.5		36.3		38.1		32.1		40.6
	12	23.1		14.2		13.3		20.2		13.3
	13–15	15.1		11.5		10.2		11.3		9.4
	>15	19.5		13.0		10.4		12.2		9.0
Civil status										
	Married	39.4		45.0		40.9		28.4		33.2
	Unmarried	50.3		33.1		35.1		56.2		36.4
	Divorced	9.6		20.0		22.1		14.6		28.6
	Widowed	0.7		1.9		1.9		0.8		1.8
Number of children <18 years										
	0	50.5		73.4		78.2		61.4		79.7
	1–2	40.5		22.7		18.7		31.6		17.8
	≥3	9.0		3.9		3.1		7.0		2.5
Parents’ occupation										
	Non-manual higher level	6.8		4.2		3.5		5.8		3.6
	Non-manual intermediate level	18.0		13.4		12.0		15.9		14.2
	Non-manual assistant	10.4		9.4		9.1		9.6		9.4
	Skilled manual	22.6		24.3		24.8		24.8		27.5
	Non-skilled manual	22.2		26.8		28.2		24.7		27.9
	Farmer	4.5		6.4		6.5		4.9		3.8
	No record	15.5		15.5		15.9		14.3		13.6
Early death of parents		5.6		2.6		2.0		8.2		3.1
Previous long-term sick leave		6.5		15.8		16.5		15.2		17.8
History of somatic disorders		79.4		84.1		83.0		85.3		85.7
History of cardiovascular diseases		4.4		12.5		19.5		6.4		10.5
History of psychiatric disorders		3.9		9.2		9.8		19.1		18.8
History of alcohol-related morbidity		1.1		4.5		5.3		6.4		15.3
History of suicide attempt		0.8		1.6		1.5		4.5		2.7

In line with our hypotheses, low control was associated with a higher risk of all-cause, CVD, and suicide mortality across three models among both men and women. Low control was robustly associated with higher alcohol-related mortality among women, but among men the association was not statistically significant in model 3 (tables 2 and 3).

**Table 2:** Hazard ratio (HR) with 95% confidence intervals (CI) of the association between trajectories of job control and job demands and all-cause and cause-specific mortality among **men**.

			All-cause		CVD		Suicide		Alcohol-related
			HR (95% CI)		HR (95% CI)		HR (95% CI)		HR (95% CI)
**Job control**									
	Model 1 ^a^								
		High	Ref		Ref		Ref		Ref
		Medium-high	1.41 (1.38–1.44)†		1.54 (1.48–1.61)†		1.74 (1.60–1.88)†		1.62 (1.45–1.82)†
		Medium-low	1.85 (1.81–1.89)†		2.23 (2.15–2.33)†		2.13 (1.96–2.31)†		2.07 (1.84–2.33)†
		Low	1.87 (1.82–1.93)†		2.21 (2.10–2.34)†		2.16 (1.94–2.40)†		2.06 (1.75–2.42)†
	Model 2 ^b^								
		High	Ref		Ref		Ref		Ref
		Medium-high	1.25 (1.23–1.28)†		1.33 (1.28–1.39)†		1.50 (1.38–1.63)†		1.30 (1.15–1.46)†
		Medium-low	1.53 (1.50–1.55)†		1.75 (1.68–1.83)†		1.74 (1.60–1.89)†		1.42 (1.25–1.61)†
		Low	1.52 (1.48–1.57)†		1.69 (1.60–1.79)†		1.76 (1.57–1.96)†		1.40 (1.19–1.66)†
	Model 3 ^c^								
		High	Ref		Ref		Ref		Ref
		Medium-high	1.14 (1.12–1.17)†		1.21 (1.16–1.26)†		1.35 (1.24–1.47)†		1.12 (0.99–1.26)
		Medium-low	1.33 (1.30–1.36)†		1.50 (1.44–1.57)†		1.49 (1.36–1.63)†		1.14 (0.99–1.30)
		Low	1.32 (1.28–1.36)†		1.45 (1.37–1.54)†		1.49 (1.33–1.68)†		1.13 (0.95–1.34)
**Job demands**									
	Model 1 ^a^								
		Low	Ref		Ref		Ref		Ref
		Medium-low	0.74 (0.72–0.75)†		0.70 (0.68–0.73)†		0.68 (0.64–0.73)†		0.66 (0.59–0.72)†
		Medium-high	0.65 (0.64–0.67)†		0.60 (0.57–0.62)†		0.56 (0.52–0.60)†		0.53 (0.47–0.60)†
		High	0.54 (0.53–0.56)†		0.45 (0.43–0.48)†		0.50 (0.45–0.57)†		0.42 (0.35–0.50)†
	Model 2 ^b^								
		Low	Ref		Ref		Ref		Ref
		Medium-low	0.82 (0.80–0.83)†		0.80 (0.77–0.83)†		0.75 (0.71–0.80)†		0.80 (0.72–0.88)†
		Medium-high	0.75 (0.74–0.77)†		0.72 (0.69–0.75)†		0.65 (0.60–0.70)†		0.69 (0.62–0.78)†
		High	0.68 (0.66–0.70)†		0.60 (0.57–0.64)†		0.64 (0.57–0.73)†		0.64 (0.54–0.76)†
	Model 3 ^c^								
		Low	Ref		Ref		Ref		Ref
		Medium-low	0.87 (0.86–0.89)†		0.87 (0.84–0.90)†		0.81 (0.76–0.87)†		0.87 (0.79–0.97)†
		Medium-high	0.83 (0.82–0.85)†		0.80 (0.77–0.83)†		0.72 (0.66–0.78)†		0.79 (0.70–0.89)†
		High	0.80 (0.78–0.83)†		0.74 (0.69–0.78)†		0.76 (0.67–0.86)†		0.81 (0.67–0.97)*

^a^ Adjusted for age. ^b^ Model 1 + adjusted for birth year, civil status, birth country, number of children, childhood socioeconomic position, previous long-term sick leave and other outcome-specific covariates. ^c^ Model 2 + education. * P<0.05 † P<0.01

**Table 3:** Hazard ratios (HR) with 95% confidence intervals (CI) of the association between trajectories of job control and job demands and all-cause and cause-specific mortality among **women**.

			All-cause		CVD		Suicide		Alcohol-related
			HR (95% CI)		HR (95% CI)		HR (95% CI)		HR (95% CI)
**Job control**									
	Model 1 ^a^								
		High	Ref		Ref		Ref		Ref
		Medium-high	1.17 (1.14–1.21)†		1.32 (1.22–1.42)†		1.33 (1.16–1.53)†		1.45 (1.16–1.83)†
		Medium-low	1.56 (1.51–1.60)†		2.16 (2.00–2.33)†		2.05 (1.78–2.35)†		2.44 (1.95–3.06)†
		Low	1.57 (1.51–1.62)†		2.33 (2.12–2.55)†		1.76 (1.48–2.09)†		2.25 (1.70–2.98)†
	Model 2 ^b^								
		High	Ref		Ref		Ref		Ref
		Medium-high	1.14 (1.11–1.17)†		1.25 (1.15–1.35)†		1.25 (1.08–1.43)†		1.38 (1.09–1.73)†
		Medium-low	1.43 (1.39–1.47)†		1.86 (1.72–2.02)†		1.69 (1.47–1.94)†		2.08 (1.66–2.63)†
		Low	1.44 (1.39–1.49)†		2.01 (1.83–2.21)†		1.47 (1.24–1.75)†		1.93 (1.45–2.56)†
	Model 3 ^c^								
		High	Ref		Ref		Ref		Ref
		Medium-high	1.06 (1.03–1.09)†		1.10 (1.01–1.19)†		1.20 (1.04–1.39)*		1.17 (0.93–1.48)
		Medium-low	1.20 (1.16–1.24)†		1.39 (1.27–1.51)†		1.54 (1.32–1.80)†		1.45 (1.13–1.85)†
		Low	1.16 (1.12–1.21)†		1.42 (1.28–1.57)†		1.32 (1.10–1.60)†		1.37 (1.05–1.80)*
**Job demands**									
	Model 1 ^a^								
		Low	Ref		Ref		Ref		Ref
		Medium-low	0.78 (0.76–0.81)†		0.67 (0.63–0.72)†		0.96 (0.82–1.12)		0.88 (0.70–1.10)
		Medium-high	0.58 (0.56–0.60)†		0.40 (0.37–0.43)†		0.62 (0.52–0.73)†		0.52 (0.41–0.68)†
		High	0.49 (0.46–0.51)†		0.28 (0.25–0.32)†		0.63 (0.50–0.78)†		0.31 (0.21–0.45)†
	Model 2 ^b^								
		Low	Ref		Ref		Ref		Ref
		Medium-low	0.80 (0.78–0.83)†		0.71 (0.66–0.76)†		1.03 (0.89–1.20)		0.93 (0.74–1.16)
		Medium-high	0.62 (0.60–0.64)†		0.45 (0.42–0.49)†		0.76 (0.64–0.90)†		0.62 (0.48–0.80)†
		High	0.55 (0.52–0.57)†		0.35 (0.31–0.39)†		0.83 (0.66–1.04)		0.40 (0.27–0.60)†
	Model 3 ^c^								
		Low	Ref		Ref		Ref		Ref
		Medium-low	0.86 (0.83–0.88)†		0.77 (0.72–0.83)†		1.08 (0.93–1.26)		1.06 (0.84–1.34)
		Medium-high	0.74 (0.72–0.77)†		0.61 (0.56–0.67)†		0.87 (0.73–1.05)		0.94 (0.71–1.25)
		High	0.73 (0.69–0.77)†		0.59 (0.51–0.68)†		1.03 (0.81–1.33)		0.87 (0.56–1.36)

^a^ Adjusted for age. ^b^ Model 1 + adjusted for birth year, civil status, birth country, number of children, childhood socioeconomic position, previous long-term sick leave and other outcome-specific covariates. ^c^ Model 2 + education. *P<0.05 †P<0.01

Compared to low demands, high demands were robustly associated with a lower risk of all outcomes among men (table 2). Similar patterns were observed among women, although high demands were not associated with suicide and alcohol-related mortality in model 3 among women (table 3).

Table 4 shows the associations between job strain categories and all-cause and cause-specific mortality. Exposures to passive jobs, in comparison with low strain jobs, were associated with higher all-cause, CVD, and suicide mortality among both men and women.

**Table 4:** Hazard ratios (HR) with 95% confidence intervals (CI) of the association between job strain categories and all-cause and cause-specific mortality by sex.

			All-cause		CVD		Suicide		Alcohol-related
			HR (95% CI)		HR (95% CI)		HR (95% CI)		HR (95% CI)
Men									
	Model 1 ^a^								
		Low strain	Ref		Ref		Ref		Ref
		Active job	0.73 (0.71–0.74)†		0.67 (0.64–0.70)†		0.62 (0.57–0.68)†		0.59 (0.52–0.66)†
		Passive job	1.39 (1.36–1.41)†		1.53 (1.47–1.58)†		1.39 (1.30–1.48)†		1.32 (1.20–1.46)†
		High strain	1.22 (1.19–1.25)†		1.30 (1.23–1.37)†		1.15 (1.04–1.28)†		1.04 (0.89–1.22)
	Model 2 ^b^								
		Low strain	Ref		Ref		Ref		Ref
		Active job	0.80 (0.79–0.82)†		0.76 (0.73–0.79)†		0.71 (0.65–0.77)†		0.72 (0.64–0.81)†
		Passive job	1.26 (1.23–1.28)†		1.34 (1.30–1.39)†		1.27 (1.19–1.36)†		1.10 (0.99–1.22)
		High strain	1.15 (1.12–1.19)†		1.20 (1.14–1.27)†		1.11 (0.99–1.23)		0.94 (0.80–1.10)
	Model 3 ^c^								
		Low strain	Ref		Ref		Ref		Ref
		Active job	0.88 (0.86–0.89)†		0.84 (0.81–0.88)†		0.77 (0.71–0.84)†		0.83 (0.73–0.94)†
		Passive job	1.19 (1.17–1.21)†		1.27 (1.22–1.31)†		1.19 (1.12–1.27)†		1.02 (0.92–1.13)
		High strain	1.10 (1.07–1.13)†		1.15 (1.09–1.21)†		1.04 (0.94–1.16)		0.89 (0.75–1.04)
Women									
	Model 1 ^a^								
		Low strain	Ref		Ref		Ref		Ref
		Active job	0.75 (0.73–0.77)†		0.62 (0.58–0.66)†		0.76 (0.67–0.86)†		0.56 (0.46–0.69)†
		Passive job	1.24 (1.22–1.27)†		1.52 (1.44–1.61)†		1.56 (1.41–1.74)†		1.48 (1.25–1.74)†
		High strain	0.93 (0.88–0.97)†		0.90 (0.80–1.02)		1.04 (0.84–1.29)		1.33 (0.98–1.81)
	Model 2 ^b^								
		Low strain	Ref		Ref		Ref		Ref
		Active job	0.79 (0.77–0.80)†		0.67 (0.62–0.71)†		0.84 (0.74–0.95)†		0.62 (0.50–0.76)†
		Passive job	1.20 (1.17–1.22)†		1.42 (1.34–1.50)†		1.42 (1.27–1.58)†		1.37 (1.16–1.62)†
		High strain	0.93 (0.89–0.97)†		0.91 (0.80–1.03)		1.04 (0.84–1.30)		1.36 (1.01–1.85)*
	Model 3 ^c^								
		Low strain	Ref		Ref		Ref		Ref
		Active job	0.89 (0.87–0.92)†		0.84 (0.78–0.91)†		0.86 (0.75–0.98)*		0.83 (0.66–1.04)
		Passive job	1.13 (1.10–1.15)†		1.30 (1.22–1.37)†		1.38 (1.23–1.54)†		1.22 (1.03–1.44)*
		High strain	0.94 (0.90–0.99)*		0.93 (0.82–1.05)		1.04 (0.84–1.30)		1.39 (1.03–1.89)*

^a^ Adjusted for age. ^b^ Model 1 + adjusted for birth year, civil status, birth country, number of children, childhood socioeconomic position, previous long-term sick leave and other outcome-specific covariates. ^c^ Model 2 + education. *P<0.05 †P<0.01

There were sex differences in other associations. Compared to low strain jobs, high strain jobs were associated with higher all-cause and CVD mortality across three models among men but not women. Passive and high strain jobs were associated with alcohol-related mortality among women but not men.

When looking at the association between job strain categories and mortality from suicide by age (supplementary table S5), the association between passive jobs and higher suicide mortality was robust in all but the oldest age group (ie, age 50–60 years).

Results from the analyses using only job exposures in 2009 were largely similar to the main results (supplementary tables S6–8).

## Discussion

In this large register-based study, we found that individuals had stable levels of job control and demands assessed using JEM. Low control and passive jobs were associated with a higher risk of all-cause, CVD, and suicide mortality among both men and women. However, high strain jobs were associated with higher all-cause and CVD mortality among men, and low control, passive jobs, and high strain jobs were associated with higher alcohol-related mortality among women. Finally, high job demands were associated with a lower risk of all outcomes, especially among men.

Our findings that low control and passive jobs were associated with higher all-cause and CVD mortality were in line with the STRESSJEM study (11, 12). In the previous meta-analyses, it was only low control that was associated with higher all-cause and coronary heart disease mortality (8). However, among the two studies included that also used JEM and tested job strain categories, one study found passive jobs associated with higher all-cause mortality (23). No studies included in the meta-analyses used JEM and tested job strain categories for coronary heart disease mortality.

In line with another meta-analysis (9) and our previous study based on the same cohort that only used job exposures in 2005 (24), low control was associated with higher suicide mortality in both sexes. While we found passive jobs to be associated with higher suicide mortality in both sexes, in the STRESSJEM (14) and our previous (24) study, the association was present only among men.

We found that the associations of low job control, passive jobs, and high strain jobs with higher alcohol-related mortality were present mainly among women. These findings may be explained by the differential drinking behavior between men and women: women are more likely to drink for negative reinforcement (eg, stress and negative affect), while men are more likely to drink for positive reinforcement (eg, stimulation) (25). This suggests that women may possibly drink more alcohol due to negative psychosocial exposures at work than men do.

Contrary to previous studies where job demands were generally not associated with mortality (8, 9, 11–14), high job demands were associated with lower risk of all outcomes in our study. This may be due to the use of JEM that capture other aspects of the occupations apart from demands. Thus, we cannot rule out the presence of residual confounding in our study.

Biological, social, and behavioral mechanisms may underly the observed associations. Low control or high strain jobs may represent stressful work scenarios. Biologically, sustained or repeated stress can lead to the dysregulation of stress responses that subsequently interrupts homeostasis, the state of steady internal conditions, in the human body (26). Consequently, the dysfunction of stress responses can affect the regulation of multiple systems in the body, and specifically of cardiovascular system (27) and the brain (28, 29). On the other hand, workers in a passive job, including low control and low demands, may experience boredom or difficulties in living up to expectations from the social context or finding self-identity, which can also be a source of stress (30). These biological and social mechanisms may partly explain the associations between low control, high strain and passive jobs and all-cause and CVD mortality.

Behaviorally, stress and boredom at work may lead to unhealthy lifestyle, such as excessive alcohol use, which can increase the risk of all-cause, CVD, and alcohol-related mortality. Finally, depression and hopelessness – feelings that there is no way to change one’s circumstances – resulting from work may contribute to suicidal behaviors and deaths (31).

The strengths of this study include the nation-wide, representative sample, which can reduce bias due to selection or attrition. Assessing psychosocial work exposures using JEM allows for objective measures and reduces reporting bias. Utilizing up to five years of occupational information and GBTM enables the consideration of hypothetical time-varying or long-term exposures to psychosocial working conditions over the years. The cause of death register provides data of high quality (32) that include all deaths during the follow-up in Sweden and enable the identification of causes of death. Furthermore, using patient registers and information of individuals’ parents allows for the control of important confounding factors, such as medical history and life-course SEP.

Several limitations of this study deserve acknowledgement. Firstly, JEM assess job exposures on the occupational level and do not capture the potential variations of individuals’ experiences or work environment within a particular occupation. In addition, GBTM is a population average approach and not optimal for finding trajectories that are less common. We observed nearly horizontal trajectories of job demands and control over the years, which suggests that most individuals stayed in a similar occupation category over the years. Thus, our approach may not necessarily capture the long-term or time-varying effect of job exposures. Indeed, our results were largely similar when using only job exposures in 2009. Nevertheless, studies conducted in Australia showed almost identical estimates of association between self-reported job control and all-cause mortality using baseline (33) and time-varying (34) measures, even though self-reported measures are more prone to fluctuations. Our findings therefore support the authors’ conclusion that baseline exposure is a reasonable proxy for time-varying measure of current exposure, which can be simplistically understood as longer-term time-weighted average exposure.

Secondly, the patient registers started from 1973 onwards, so medical histories prior to 1973 are not available. Additionally, diagnoses of somatic and psychiatric disorders in the registers tend to be more severe cases including those who get hospital or specialized treatment and miss milder untreated cases or cases treated in primary care. Furthermore, detailed lifestyle factors are not available in the register-based study. All these might have contributed to residual confounding in the results. Thirdly, medical conditions prior to baseline could partially be the consequence of previous exposures to negative psychosocial working conditions. Therefore, the adjustment of medical history might have resulted in an underestimation of associations in our study. Nevertheless, it would not influence the conclusion drawn from our results. Further, using father’s occupation to represent individuals’ early-life SEP could have led to misclassification for some individuals whose mothers provided the main economic support to their families. Finally, we acknowledge that psychosocial working conditions consist of multiple aspects beyond Karasek’s job demand–control model. A few other perspectives are the effort–reward imbalance model, job insecurity, and unwanted conduct at work including workplace bullying. Future studies on psychosocial working conditions and mortality are encouraged to adopt a holistic approach where various perspectives are considered simultaneously.

In conclusion, adverse psychosocial working conditions characterized by low job control, passive jobs, and high strain jobs are related to higher all-cause mortality and mortality due to several specific causes, though these patterns vary to some extent between men and women. These findings add to the existing literature regarding the impact of poor psychosocial working conditions on individuals’ health. A synthesis of systematic reviews showed that the effects of interventions on psychosocial working conditions, such as increasing job control, tend to be related to less absenteeism and increased financial benefit and productivity or performance (35). It would be important to further examine whether such interventions have effect on mortality or specific causes of death.

## Supplementary material

Supplementary material
