# Supplementary material for: Trajectories of psychosocial working conditions and all-cause and cause-specific mortality: a Swedish register-based cohort study
Source: Scand J Work Environ Health. 2023 Sep 29;49(7):496–505. doi: 10.5271/sjweh.4111 (PMC10830330; doi:10.5271/sjweh.4111)
Supplement: Supplementary material [file SJWEH-49-496-S001.pdf]

**Trajectories of psychosocial working conditions and all-cause and cause-specific mortality: a Swedish register-based cohort study[1]**

by Kuan-Yu Pan, PhD,[2] Melody Almroth, PhD, Alicia Nevriana, PhD, Tomas Hemmingsson, PhD, Katarina Kjellberg, PhD, Daniel Falkstedt, PhD

1. *Supplementary Material*
2. *Kuan-Yu Pan, PhD, Unit of Occupational Medicine, Institute of Environmental Medicine, Karolinska Institutet, Solnavägen 4, 113 65 Stockholm, Sweden. [E-mail: kuan-yu.pan@ki.se]*

Table S1. Translated items used for the decision authority, skill discretion and psychological demands in the Job Exposure Matrices

| Dimension | Question | Answer alternatives |
|---|---|---|
| Job control | | |
| Decision authority | Can you partially decide when tasks should be done? | Never, mostly not, mostly, always |
| | Do you have the opportunity to decide your own work pace? | Not at all, occasionally, roughly ¼ of the time, half of the time, roughly ¾ of the time, almost all the time |
| | Can you take short breaks to talk pretty much any time? | Not at all, occasionally, roughly ¼ of the time, half of the time, roughly ¾ of the time, almost all the time |
| | Are you ever involved in deciding how your work is organized? | Never, mostly not, mostly, always |
| Skill discretion | Is there any apprenticeship or introductory training required at the workplace (besides education or course) before you can do your job? | No, only a few days, a few weeks, a few months, ½ year, 1 year, 2 years, 3 years, 4 years or more |
| | Does the work require you to repeat the same work steps many times an hour? | Not at all, occasionally, roughly ¼ of the time, half of the time, roughly ¾ of the time, almost all the time |
| | Do you spend quite some time during the day trying to understand or solving difficult problems? | Occasionally, roughly ¼ of the time, half of the time, roughly ¾ of the time, almost all the time |
| | Does the work offer you the possibility to learn something new and to develop in this occupation? | Not at all, a few days per month, one day per week, a few days per week, every day |
| Job demands | | |
| Psychological demands | Are you sometimes so stressed that you do not have time to talk about or even think about something besides work? | Not at all, occasionally, roughly ¼ of the time, half of the time, roughly ¾ of the time, almost all the time |
| | Do you sometimes have so much to do that you have to work during lunch, work overtime, or take work home? | Not at all, a few days per month, one day per week, a few days per week, every day |
| | Does your work require all your attention and concentration? | Not at all, occasionally, roughly ¼ of the time, half of the time, roughly ¾ of the time, almost all the time |

Table S2. Diagnostic of trajectory models of job control and job demands by number of groups and sex

| Group | Estimated proportion from the trajectory model* | Proportion by posterior probability-based classification* | Average posterior probability | Odds of correct classification |
|---|---|---|---|---|
| **3 groups** | | | | |
| **Job control – men (BIC: -22459450.8)** | | | | |
| 1 | 0.31 | 0.31 | 0.98 | 108.1 |
| 2 | 0.40 | 0.40 | 0.97 | 45.6 |
| 3 | 0.29 | 0.29 | 0.98 | 126.6 |
| **Job demands – men (BIC: -11389122.4)** | | | | |
| 1 | 0.14 | 0.14 | 0.98 | 267.5 |
| 2 | 0.51 | 0.51 | 0.97 | 31.1 |
| 3 | 0.35 | 0.35 | 0.98 | 76.4 |
| **Job control – women (BIC: -21436342.9)** | | | | |
| 1 | 0.38 | 0.39 | 0.99 | 119.6 |
| 2 | 0.41 | 0.41 | 0.97 | 51.0 |
| 3 | 0.21 | 0.20 | 0.98 | 156.2 |
| **Job demands – women (BIC: -14851036.6)** | | | | |
| 1 | 0.15 | 0.15 | 0.98 | 427.5 |
| 2 | 0.74 | 0.74 | 0.99 | 48.8 |
| 3 | 0.11 | 0.11 | 0.98 | 353.4 |
| **4 groups** | | | | |
| **Job control – men (BIC: -20694973.4)** | | | | |
| 1 | 0.09 | 0.09 | 0.95 | 183.6 |
| 2 | 0.29 | 0.29 | 0.96 | 58.7 |
| 3 | 0.34 | 0.34 | 0.96 | 40.4 |
| 4 | 0.28 | 0.28 | 0.98 | 120.6 |
| **Job demands – men (BIC: -9667622.8)** | | | | |
| 1 | 0.09 | 0.09 | 0.98 | 437.2 |
| 2 | 0.24 | 0.24 | 0.95 | 56.2 |
| 3 | 0.38 | 0.38 | 0.95 | 28.9 |
| 4 | 0.29 | 0.29 | 0.97 | 74.2 |
| **Job control – women (BIC: -19766366.9)** | | | | |
| 1 | 0.11 | 0.11 | 0.96 | 183.2 |
| 2 | 0.33 | 0.33 | 0.97 | 64.1 |
| 3 | 0.38 | 0.38 | 0.94 | 27.8 |
| 4 | 0.18 | 0.18 | 0.97 | 147.3 |
| **Job demands – women (BIC: -12350346.5)** | | | | |
| 1 | 0.08 | 0.08 | 0.98 | 570.4 |
| 2 | 0.27 | 0.27 | 0.94 | 42.9 |
| 3 | 0.57 | 0.57 | 0.98 | 29.1 |
| 4 | 0.08 | 0.08 | 0.98 | 500.6 |
| **5 groups** | | | | |
| **Job control – men (BIC: -19778281.3)** | | | | |
| 1 | 0.03 | 0.03 | 0.96 | 829.6 |
| 2 | 0.19 | 0.19 | 0.95 | 86.8 |
| 3 | 0.23 | 0.23 | 0.93 | 44.2 |
| 4 | 0.28 | 0.28 | 0.96 | 53.5 |

| | | | | |
|---|---|---|---|---|
| 5 | 0.26 | 0.26 | 0.98 | 118.1 |
| **Job demands – men (BIC: -8774715.7)** | | | | |
| 1 | 0.08 | 0.08 | 0.98 | 501.5 |
| 2 | 0.24 | 0.24 | 0.95 | 65.6 |
| 3 | 0.33 | 0.33 | 0.95 | 34.7 |
| 4 | 0.25 | 0.25 | 0.94 | 47.5 |
| 5 | 0.10 | 0.10 | 0.95 | 162.7 |
| **Job control – women (BIC: -18804651.4)** | | | | |
| 1 | 0.10 | 0.10 | 0.97 | 323.6 |
| 2 | 0.30 | 0.30 | 0.97 | 87.2 |
| 3 | 0.30 | 0.30 | 0.95 | 42.3 |
| 4 | 0.16 | 0.16 | 0.94 | 74.2 |
| 5 | 0.14 | 0.14 | 0.98 | 266.3 |
| **Job demands – women (BIC: -11268577.5)** | | | | |
| 1 | 0.06 | 0.07 | 0.99 | 976.4 |
| 2 | 0.20 | 0.20 | 0.96 | 99.7 |
| 3 | 0.36 | 0.36 | 0.94 | 26.1 |
| 4 | 0.32 | 0.31 | 0.94 | 33.3 |
| 5 | 0.06 | 0.06 | 0.98 | 827.6 |

*There should be sufficient sample size in each identified group (more than 5%) and close correspondence between these two columns

Table S3. ICD codes for somatic disorders, psychiatric disorders, cardiovascular diseases, alcohol-related morbidity, and suicide

| | ICD-10 | ICD-9 | ICD-8 |
|---|---|---|---|
| Somatic disorders | All codes in the patient registers excluding F, O, P, Q codes | All codes in the patient registers excluding 29, 30, 31, 63, 64, 65, 66, 67, 74, 75, 76, 77 | All codes in the patient registers excluding 29, 30, 31, 63, 64, 65, 66, 67, 74, 75, 76, 77 |
| Psychiatric disorders | F01-F99 | 29-31 | 29-31 |
| Cardiovascular diseases | I00-I99 | 39-45, | 39-45 |
| Suicide (attempt) | X60-X84, Y10-Y34 | 95 | 95 |
| Alcohol-related morbidity | F10, K70, T51, X65, Y90, Y91, E24.4, G31.2, G62.1, G72.1, I42.6, K29.2, K85.2, K86.0, O35.4, R78.0, Y57.3, Z50.2, Z71.4, Z72.1 | 291, 303, 357, 425, 535, 571, 790, 977, 980 | 261, 262, 291, 303, 571, 980 |

Table S4. Covariates included in model 3 by cause of death

| | All-cause | CVD | Suicide | Alcohol-related |
|---|---|---|---|---|
| Age | x | x | x | x |
| Birth year | x | x | x | x |
| Birth country | x | x | x | x |
| Sex | x | x | x | x |
| Education | x | x | x | x |
| Civil status | x | x | x | x |
| Number of children | x | x | x | x |
| Childhood SEP | x | x | x | x |
| Early death of parents | x | x | | x |
| Previous Long-term sick leave | x | x | x | x |
| History of somatic disorders | x | | | |
| History of psychiatric disorders | x | | x | |
| History of CVD | | x | | |
| Previous suicide attempt | | | x | |
| History of alcohol morbidity | | | | x |

SEP, Socioeconomic position; CVD, Cardiovascular diseases

Table S5. Hazard ratios (HRs) with 95% confidence intervals (CIs) of the association between job strain categories and suicide, by age groups

| | Age 16-29 | Age 30-39 | Age 40-49 | Age 50-60 |
|---|---|---|---|---|
| | HR (95% CI) | HR (95% CI) | HR (95% CI) | HR (95% CI) |
| Model 1 | | | | |
| Low strain | Ref | Ref | Ref | Ref |
| Active job | 0.60 (0.50-0.72)† | 0.66 (0.58-0.76)† | 0.69 (0.61-0.78)† | 0.64 (0.56-0.72)† |
| Passive job | 1.67 (1.50-1.87)† | 1.63 (1.45-1.82)† | 1.33 (1.20-1.47)† | 1.12 (1.00-1.26)* |
| High strain | 1.10 (0.91-1.34) | 1.35 (1.11-1.63)† | 1.12 (0.94-1.33) | 1.02 (0.85-1.23) |
| Model 2 | | | | |
| Low strain | Ref | Ref | Ref | Ref |
| Active job | 0.64 (0.53-0.77)† | 0.74 (0.65-0.85)† | 0.80 (0.71-0.90)† | 0.72 (0.63-0.81)† |
| Passive job | 1.59 (1.43-1.78)† | 1.44 (1.28-1.61)† | 1.18 (1.06-1.31)† | 1.00 (0.89-1.12) |
| High strain | 1.11 (0.91-1.35) | 1.27 (1.04-1.54)* | 1.05 (0.88-1.25) | 0.98 (0.82-1.18) |
| Model 3 | | | | |
| Low strain | Ref | Ref | Ref | Ref |
| Active job | 0.72 (0.59-0.87)† | 0.82 (0.71-0.95)† | 0.85 (0.75-0.97)* | 0.70 (0.61-0.80)† |
| Passive job | 1.43 (1.28-1.60)† | 1.31 (1.17-1.48)† | 1.12 (1.01-1.25)* | 1.00 (0.89-1.13) |
| High strain | 1.06 (0.97-1.29) | 1.19 (0.98-1.45) | 1.01 (0.85-1.20) | 0.98 (0.81-1.17) |

Job strain categories based on 4 groups of job control and 4 groups of job demands

Model 1 adjusting for age and sex

Model 2 adjusting for age, sex, birth year, civil status, birth country, number of children, childhood socioeconomic position, previous long-term sick leave, history of psychiatric disorders, and previous suicide attempt

Model 3 adjusting for age, sex, birth year, civil status, birth country, number of children, childhood socioeconomic position, previous long-term sick leave, history of psychiatric disorders, previous suicide attempt, and education

*p<0.05 †p<0.01

Table S6. Hazard ratios (HRs) with 95% confidence intervals (CIs) of the association between job control and job demands in 2009 and all-cause and cause-specific mortality in men

| | **All-cause** | **CVD** | **Suicide** | **Alcohol-related** |
|---|---|---|---|---|
| **Job control** | HR (95% CI) | HR (95% CI) | HR (95% CI) | HR (95% CI) |
| Model 1 | | | | |
| High | Ref | Ref | Ref | Ref |
| Medium-high | 1.41 (1.38-1.44)† | 1.54 (1.48-1.61)† | 1.74 (1.60-1.88)† | 1.64 (1.46-1.84)† |
| Medium-low | 1.85 (1.81-1.89)† | 2.24 (2.15-2.33)† | 2.13 (1.96-2.31)† | 2.09 (1.85-2.34)† |
| Low | 1.88 (1.83-1.93)† | 2.22 (2.10-2.34)† | 2.16 (1.95-2.41)† | 2.07 (1.76-2.43)† |
| Model 2 | | | | |
| High | Ref | Ref | Ref | Ref |
| Medium-high | 1.25 (1.23-1.28)† | 1.34 (1.28-1.39)† | 1.50 (1.38-1.63)† | 1.31 (1.16-1.47)† |
| Medium-low | 1.53 (1.50-1.57)† | 1.76 (1.68-1.83)† | 1.73 (1.59-1.89)† | 1.43 (1.26-1.62)† |
| Low | 1.52 (1.48-1.57)† | 1.69 (1.60-1.79)† | 1.75 (1.57-1.96)† | 1.41 (1.20-1.67)† |
| Model 3 | | | | |
| High | Ref | Ref | Ref | Ref |
| Medium-high | 1.14 (1.12-1.17)† | 1.21 (1.16-1.26)† | 1.35 (1.24-1.47)† | 1.13 (0.99-1.27) |
| Medium-low | 1.33 (1.30-1.36)† | 1.50 (1.44-1.57)† | 1.48 (1.35-1.63)† | 1.15 (1.01-1.31) |
| Low | 1.32 (1.28-1.36)† | 1.45 (1.37-1.54)† | 1.49 (1.33-1.68)† | 1.14 (0.96-1.35) |
| **Job demands** | | | | |
| Model 1 | | | | |
| Low | Ref | Ref | Ref | Ref |
| Medium-low | 0.74 (0.72-0.75)† | 0.71 (0.68-0.73)† | 0.68 (0.64-0.73)† | 0.66 (0.59-0.73)† |
| Medium-high | 0.65 (0.64-0.67)† | 0.60 (0.57-0.62)† | 0.56 (0.51-0.60)† | 0.53 (0.47-0.60)† |
| High | 0.54 (0.53-0.56)† | 0.45 (0.43-0.48)† | 0.50 (0.45-0.56)† | 0.42 (0.36-0.50)† |
| Model 2 | | | | |
| Low | Ref | Ref | Ref | Ref |
| Medium-low | 0.82 (0.80-0.83)† | 0.80 (0.77-0.83)† | 0.75 (0.70-0.80)† | 0.80 (0.72-0.89)† |
| Medium-high | 0.75 (0.74-0.77)† | 0.72 (0.69-0.74)† | 0.65 (0.60-0.70)† | 0.69 (0.62-0.78)† |
| High | 0.68 (0.66-0.70)† | 0.60 (0.57-0.64)† | 0.64 (0.57-0.72)† | 0.64 (0.54-0.77)† |
| Model 3 | | | | |
| Low | Ref | Ref | Ref | Ref |
| Medium-low | 0.87 (0.86-0.89)† | 0.87 (0.84-0.90)† | 0.81 (0.76-0.86)† | 0.88 (0.79-0.97)† |

| | | | | |
|---|---|---|---|---|
| Medium-high | 0.83 (0.82-0.85)† | 0.80 (0.77-0.83)† | 0.71 (0.66-0.77)† | 0.79 (0.70-0.89)† |
| High | 0.80 (0.78-0.83)† | 0.73 (0.69-0.78)† | 0.76 (0.67-0.86)† | 0.82 (0.68-0.98)* |

Model 1 adjusting for age

Model 2 adjusting for age, birth year, civil status, birth country, number of children, childhood socioeconomic position, previous long-term sick leave and other outcome-specific covariates

Model 3 adjusting for age, birth year, civil status, birth country, number of children, childhood socioeconomic position, previous long-term sick leave, other outcome-specific covariates, and education *p<0.05 †p<0.01

Table S7. Hazard ratios (HRs) with 95% confidence intervals (CIs) of the association between job control and job demands in 2009 and all-cause and cause-specific mortality in women

| | **All-cause** | **CVD** | **Suicide** | **Alcohol-related** |
|---|---|---|---|---|
| **Job control** | HR (95% CI) | HR (95% CI) | HR (95% CI) | HR (95% CI) |
| Model 1 | | | | |
| High | Ref | Ref | Ref | Ref |
| Medium-high | 1.17 (1.14-1.21)† | 1.32 (1.22-1.42)† | 1.33 (1.15-1.52)† | 1.45 (1.16-1.83)† |
| Medium-low | 1.56 (1.51-1.60)† | 2.16 (2.00-2.33)† | 2.04 (1.78-2.34)† | 2.44 (1.95-3.06)† |
| Low | 1.57 (1.51-1.63)† | 2.33 (2.12-2.56)† | 1.75 (1.48-2.08)† | 2.23 (1.68-2.96)† |
| Model 2 | | | | |
| High | Ref | Ref | Ref | Ref |
| Medium-high | 1.14 (1.11-1.17)† | 1.25 (1.15-1.35)† | 1.24 (1.08-1.43)† | 1.38 (1.09-1.73)† |
| Medium-low | 1.43 (1.39-1.47)† | 1.86 (1.72-2.02)† | 1.68 (1.46-1.94)† | 2.08 (1.65-2.63)† |
| Low | 1.44 (1.39-1.49)† | 2.01 (1.83-2.21)† | 1.47 (1.23-1.75)† | 1.91 (1.43-2.54)† |
| Model 3 | | | | |
| High | Ref | Ref | Ref | Ref |
| Medium-high | 1.06 (1.03-1.09)† | 1.10 (1.01-1.19)† | 1.20 (1.04-1.39)* | 1.17 (0.93-1.48) |
| Medium-low | 1.20 (1.16-1.24)† | 1.39 (1.27-1.51)† | 1.53 (1.32-1.79)† | 1.44 (1.13-1.85)† |
| Low | 1.16 (1.12-1.21)† | 1.42 (1.29-1.57)† | 1.32 (1.09-1.59)† | 1.23 (0.91-1.66) |
| **Job demands** | | | | |
| Model 1 | | | | |
| Low | Ref | Ref | Ref | Ref |
| Medium-low | 0.78 (0.76-0.81)† | 0.67 (0.63-0.72)† | 0.96 (0.83-1.12) | 0.87 (0.70-1.10) |
| Medium-high | 0.58 (0.56-0.60)† | 0.40 (0.37-0.43)† | 0.62 (0.52-0.74)† | 0.52 (0.41-0.68)† |
| High | 0.48 (0.46-0.51)† | 0.28 (0.25-0.32)† | 0.63 (0.51-0.79)† | 0.31 (0.21-0.45)† |
| Model 2 | | | | |
| Low | Ref | Ref | Ref | Ref |
| Medium-low | 0.80 (0.78-0.83)† | 0.70 (0.66-0.76)† | 1.03 (0.89-1.20) | 0.93 (0.74-1.16) |
| Medium-high | 0.62 (0.60-0.64)† | 0.45 (0.42-0.49)† | 0.76 (0.64-0.90)† | 0.62 (0.48-0.80)† |
| High | 0.55 (0.52-0.57)† | 0.35 (0.30-0.39)† | 0.83 (0.66-1.04) | 0.40 (0.27-0.60)† |
| Model 3 | | | | |
| Low | Ref | Ref | Ref | Ref |
| Medium-low | 0.86 (0.83-0.88)† | 0.77 (0.72-0.83)† | 1.09 (0.93-1.27) | 1.06 (0.84-1.34) |

| | | | | |
|---|---|---|---|---|
| Medium-high | 0.74 (0.72-0.77)† | 0.61 (0.55-0.67)† | 0.88 (0.73-1.05) | 0.95 (0.71-1.25) |
| High | 0.73 (0.69-0.77)† | 0.59 (0.51-0.68)† | 1.04 (0.81-1.34) | 0.87 (0.56-1.37) |

Model 1 adjusting for age

Model 2 adjusting for age, birth year, civil status, birth country, number of children, childhood socioeconomic position, previous long-term sick leave and other outcome-specific covariates

Model 3 adjusting for age, birth year, civil status, birth country, number of children, childhood socioeconomic position, previous long-term sick leave, other outcome-specific covariates, and education *p<0.05 †p<0.01

Table S8. Hazard ratios (HRs) with 95% confidence intervals (CIs) of the association between job strain categories in 2009 and all-cause and cause-specific mortality by sex

| | **All-cause** | **CVD** | **Suicide** | **Alcohol-related** |
|---|---|---|---|---|
| **Men** | HR (95% CI) | HR (95% CI) | HR (95% CI) | HR (95% CI) |
| Model 1 | | | | |
| Low strain | Ref | Ref | Ref | Ref |
| Active job | 0.73 (0.71-0.74)† | 0.67 (0.64-0.70)† | 0.62 (0.57-0.67)† | 0.59 (0.52-0.66)† |
| Passive job | 1.39 (1.36-1.41)† | 1.53 (1.47-1.58)† | 1.38 (1.30-1.48)† | 1.32 (1.20-1.46)† |
| High strain | 1.22 (1.19-1.25)† | 1.30 (1.23-1.37)† | 1.15 (1.04-1.28)† | 1.05 (0.90-1.23) |
| Model 2 | | | | |
| Low strain | Ref | Ref | Ref | Ref |
| Active job | 0.80 (0.79-0.82)† | 0.76 (0.73-0.79)† | 0.71 (0.65-0.77)† | 0.72 (0.64-0.81)† |
| Passive job | 1.26 (1.23-1.28)† | 1.34 (1.30-1.39)† | 1.27 (1.19-1.36)† | 1.10 (0.99-1.22) |
| High strain | 1.15 (1.12-1.19)† | 1.20 (1.14-1.27)† | 1.10 (0.99-1.23) | 0.95 (0.81-1.11) |
| Model 3 | | | | |
| Low strain | Ref | Ref | Ref | Ref |
| Active job | 0.87 (0.86-0.89)† | 0.84 (0.81-0.88)† | 0.77 (0.70-0.83)† | 0.83 (0.73-0.94)† |
| Passive job | 1.19 (1.16-1.21)† | 1.27 (1.22-1.31)† | 1.19 (1.11-1.27)† | 1.02 (0.92-1.13) |
| High strain | 1.10 (1.07-1.13)† | 1.15 (1.09-1.21)† | 1.04 (0.94-1.15) | 0.89 (0.76-1.05) |
| **Women** | | | | |
| Model 1 | | | | |
| Low strain | Ref | Ref | Ref | Ref |
| Active job | 0.75 (0.73-0.77)† | 0.62 (0.58-0.66)† | 0.77 (0.68-0.87)† | 0.56 (0.46-0.69)† |
| Passive job | 1.24 (1.22-1.27)† | 1.52 (1.44-1.61)† | 1.57 (1.41-1.74)† | 1.47 (1.25-1.73)† |
| High strain | 0.93 (0.88-0.97)† | 0.90 (0.80-1.02) | 1.04 (0.83-1.29) | 1.33 (0.98-1.81) |
| Model 2 | | | | |
| Low strain | Ref | Ref | Ref | Ref |
| Active job | 0.79 (0.77-0.81)† | 0.67 (0.62-0.71)† | 0.84 (0.75-0.96)† | 0.62 (0.50-0.76)† |
| Passive job | 1.20 (1.17-1.23)† | 1.42 (1.34-1.50)† | 1.42 (1.28-1.58)† | 1.37 (1.16-1.61)† |
| High strain | 0.93 (0.89-0.98)† | 0.91 (0.80-1.03) | 1.04 (0.84-1.30) | 1.36 (1.00-1.85)* |
| Model 3 | | | | |
| Low strain | Ref | Ref | Ref | Ref |
| Active job | 0.89 (0.87-0.92)† | 0.84 (0.78-0.91)† | 0.86 (0.75-0.99)* | 0.83 (0.66-1.05) |

| | | | | |
|---|---|---|---|---|
| Passive job | 1.13 (1.10-1.15)† | 1.30 (1.22-1.37)† | 1.38 (1.24-1.54)† | 1.21 (1.02-1.43)* |
| High strain | 0.94 (0.90-0.99)* | 0.93 (0.82-1.05) | 1.04 (0.83-1.29) | 1.40 (1.03-1.89)* |

Model 1 adjusting for age

Model 2 adjusting for age, birth year, civil status, birth country, number of children, childhood socioeconomic position, previous long-term sick leave and other outcome-specific covariates

Model 3 adjusting for age, birth year, civil status, birth country, number of children, childhood socioeconomic position, previous long-term sick leave, other outcome-specific covariates, and education

*p<0.05 †p<0.01

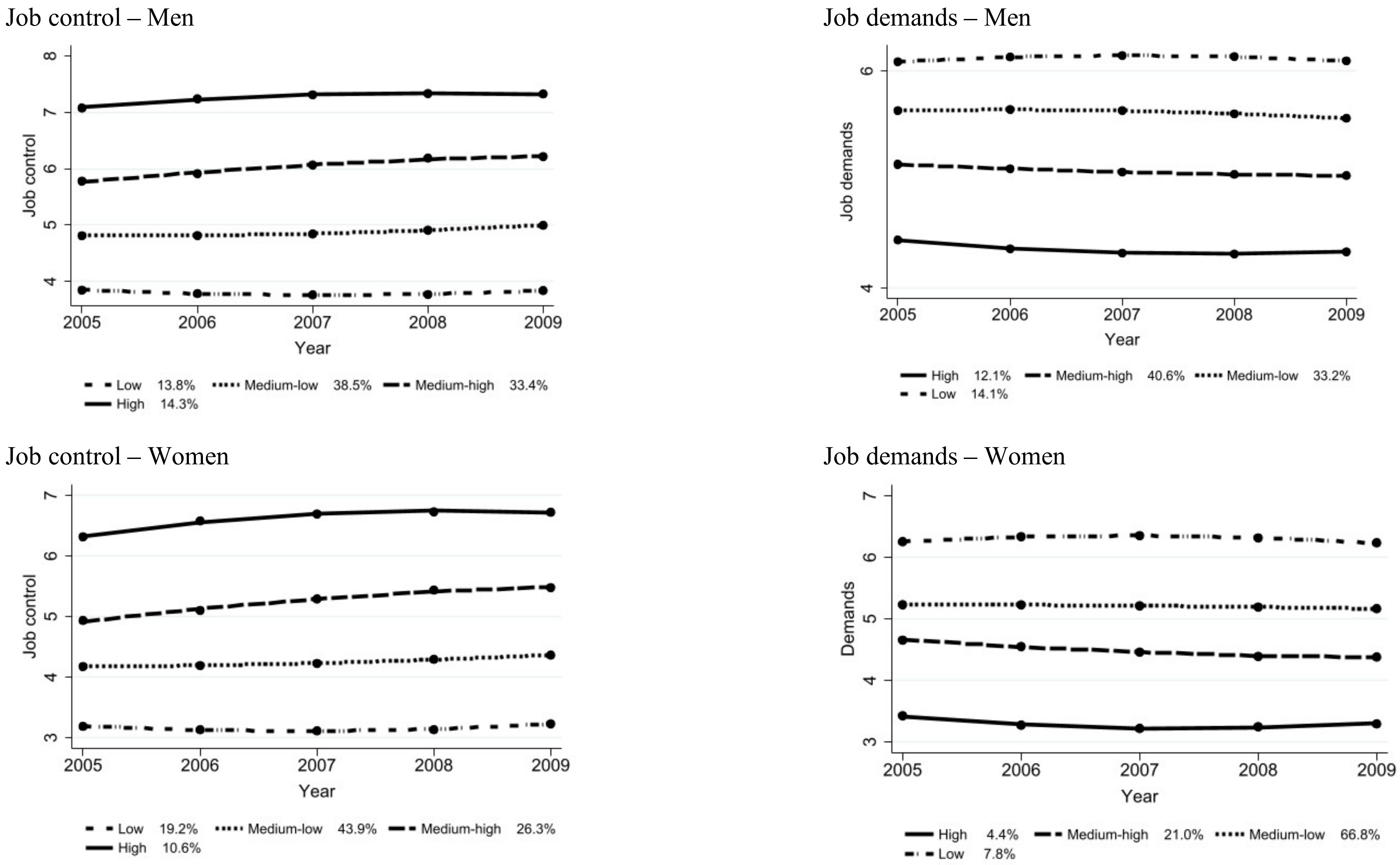


Figure S1. Trajectories of job control and job demands (2005-2009) in the Swedish working population aged below 30 in 2005 by sex

A higher score of job control means a higher level of job control; A higher score of job demands means a lower level of job demands
